# Predicting postoperative delirium after cardiovascular surgeries from preoperative portable electroencephalography oscillations

**DOI:** 10.3389/fpsyt.2023.1287607

**Published:** 2023-11-14

**Authors:** Masahiro Hata, Yuki Miyazaki, Chie Nagata, Hirotada Masuda, Tamiki Wada, Shun Takahashi, Ryouhei Ishii, Shigeru Miyagawa, Manabu Ikeda, Takayoshi Ueno

**Affiliations:** ^1^Department of Psychiatry, Osaka University Graduate School of Medicine, Osaka, Japan; ^2^Division of Health Sciences, Osaka University Graduate School of Medicine, Osaka, Japan; ^3^Department of Cardiovascular Surgery, Osaka University Graduate School of Medicine, Osaka, Japan; ^4^Department of Occupational Therapy, Graduate School of Rehabilitation Science, Osaka Metropolitan University, Osaka, Japan; ^5^Clinical Research and Education Center, Asakayama General Hospital, Osaka, Japan; ^6^Department of Neuropsychiatry, Wakayama Medical University, Wakayama, Japan

**Keywords:** EEG, delirium, machine learning, power spectrum density, cardiovascular surgery

## Abstract

**Introduction:**

Postoperative delirium (POD) is common and life-threatening, however, with intensive interventions, a potentially preventable clinical syndrome. Although electroencephalography (EEG) is a promising biomarker of delirium, standard 20-leads EEG holds difficulties for screening usage in clinical practice.

**Objective:**

We aimed to develop an accurate algorithm to predict POD using EEG data obtained from portable device.

**Methods:**

We recruited 128 patients who underwent scheduled cardiovascular surgery. Cognitive function assessments were conducted, and portable EEG recordings were obtained prior to surgery.

**Results:**

Among the patients, 47 (36.7%) patients with POD were identified and they did not significantly differ from patients without POD in sex ratio, age, cognitive function, or treatment duration of intensive care unit. However, significant differences were observed in the preoperative EEG power spectrum densities at various frequencies, especially gamma activity, between patients with and without POD. POD was successfully predicted using preoperative EEG data with a machine learning algorithm, yielding accuracy of 86% and area under the receiver operating characteristic curve of 0.93.

**Discussion:**

This study provides new insights into the objective and biological vulnerability to delirium. The developed algorithm can be applied in general hospitals without advanced equipment and expertise, thereby enabling the reduction of POD occurrences with intensive interventions for high-risk patients.

## 1. Introduction

Delirium is a common but life-threatening clinical syndrome in older persons (1) and is defined as acute and fluctuating neuropsychiatric disturbances of attention and consciousness with cognitive deficits (2). Delirium often develops along with acute illness, surgery, or hospitalization and can lead to a series of events that cause loss of independence, increased morbidity and mortality, institutionalization, and high medical costs (1). It is estimated that more than 2.6 million adults aged 65 years and older develop delirium each year in the United States, representing more than $164 billion in annual healthcare costs (3). Delirium adversely affects the daily functioning and quality of life of patients, and has important social implications for individuals, families, communities, and the healthcare system as a whole (1).

With regard to delirium, postoperative delirium (POD) is a common and severe but potentially preventable syndrome in older individuals (4). For pharmacological prevention, preoperative measures can be taken, such as adjusting tricyclic antidepressants, urological analgesics, benzodiazepines, and other drugs with strong anticholinergic effects (4, 5). On the other hand, as a nonpharmacological prevention, a combination of interventions, such as staff education, early mobilization, pain control, reorientation, sleep–wake cycle preservation, and optimization of hydration and nutrition, decreased the incidence of POD by 44% (4, 5). Thus, if patients at high risk for POD can be predicted in advance, the focused implementation of these interventions can effectively prevent the onset of delirium.

Electroencephalography (EEG) is a useful technique to capturing the neurophysiological characteristics of delirium (6, 7). It has been suggested that vulnerability to delirium prior to its onset can be captured using EEG (8). EEG oscillations can be affected by several surgical factors, such as anesthesia (9), hypothermia (10), and ischemia (11); however, these factors do not affect EEG oscillations prior to surgery. Therefore, the preoperative assessment offers the potential to accurately capture inherent vulnerability independently of various factors that influence EEG measurements (8). Despite these advantages, studies on the prediction of delirium using preoperative EEG are limited. Although EEG is a promising technique to identify the vulnerability of patients to delirium, several limitations preclude its routine use for the screening of large numbers of hospitalized patients. Standard 20-lead EEG devices are generally large and expensive, which impedes easy access. Additionally, experienced technicians spend considerable time correctly placing and recording 20 leads, and the data must be interpreted by an expert neurophysiologist. This leads to clinical delays in initiating appropriate treatment (12). Owing to these limitations, there is a need for EEG devices that can record data anywhere in a quick, simple manner and can automatically interpret the data without the specialists’ assessment.

In this study, we aimed to develop a precise algorithm to automatically predict the onset of POD from preoperative EEG oscillations, measured quickly and simply using a portable device. For ease of use in actual clinical practice, our study focused exclusively on developing a prediction model for postoperative delirium using EEG data as the sole modality, without considering other clinical parameters. By identifying patients at high risk of developing delirium, the targeted implementation of preventive measures is expected to prevent POD and reduce its impact on individuals, families, communities, and the healthcare system.

## 2. Materials and methods

### 2.1. Study patients

Patients aged ≥40 years who were hospitalized and underwent scheduled cardiovascular surgeries at Osaka University Hospital between November 2021 and November 2022 were recruited. All patients were of Asian race/ethnicity. Only patients aged 40 years or older we included because a previous study (13) reported very few cases of delirium after cardiac surgery among patients ≤40 years of age. We included patients undergoing a median sternotomy or thoracotomy approach (minimally invasive cardiac surgery), which included coronary artery bypass grafting with or without cardiopulmonary bypass, all types of valvular surgery except transcatheter aortic valve implantation, ascending aorta replacement, and open-heart surgery for adult congenital heart disease. The exclusion criteria were as follows: (1) patients with preoperative delirium, (2) patients diagnosed with dementia, (3) patients who required intubation for 4 or more days postoperatively, (4) patients who had a cerebral hemorrhage or stroke within 7 days postoperatively, and (5) patients who required reoperation within 7 days postoperatively. Prior to enrollment, we explained the utilization of their clinical data for this research to all patients and obtained their written informed consent. This study was approved by the ethics committee of Osaka University Hospital (approval number: 21185-3) and registered in the UMIN Clinical Trial Registry (UMIN 000049390).

### 2.2. Clinical assessment and diagnosis of delirium

Demographic and clinical characteristics, such as age, sex, usage of benzodiazepines or anticholinergics, and surgical procedures were obtained from the patients’ medical records. Benzodiazepines or anticholinergics users were identified at their admission to our hospital based on their prescription information. Anticholinergic uses were defined as users of prednisolone, theophylline, digoxin, furosemide, nifedipine, or H2 receptor antagonists, which had been reported to have strong anticholinergic effects in the study using radioreceptor assays (14). Surgical procedures were included as follows: Coronary Artery Bypass Graft (CABG) for coronary artery disease, Valve-surgery (Valve) for valvular disease, and Graft Replacement (GR) for aortic aneurysm. In addition, we assessed their state of consciousness and baseline cognitive function using the Mini-Cog (15). The Mini-cog was developed as a brief test to identify patients with dementia, and its diagnostic value is not influenced by education or language (15). All study participants were admitted to the intensive care unit (ICU) of our hospital immediately after surgery.

The patients’ medical records were reviewed daily, and the same examiner who evaluated the patients’ preoperative condition visited them for postoperative assessments. We closely monitored the patients’ level of consciousness, especially changes from the preoperative state, and asked them about their orientation to time and place, visual and auditory hallucinations, and sleep status. In addition, we elicited their recollection of their experience in the ICU, encouraged them to recall the postoperative course, and checked for any discrepancies in the patients’ medical records. Delirium is characterized by diurnally fluctuating disturbance in cognition and consciousness (16). Considering the diurnal variability in delirium, visiting hours were set at 3:30 p.m. and 6:30 p.m. The onset of delirium was diagnosed by trained psychiatrists (M. H. and Y. M.) based on the DSM-V criteria (2) by scrutinizing the medical records and assessing the patients. The research team, which included two psychiatrists, shared information to determine the final diagnosis of delirium. The observation period in this study was set up to 7 days postoperatively.

### 2.3. Portable EEG device

We utilized a multi-channel patch-type EEG sensor named “HARU-1,” which includes a wireless sensing device and a disposable electrode sheet (Supplementary Figure S1). The device has received approval for medical use from the Pharmaceutical and Medical Agency of Japan and has been reviewed for its ability to measure EEG data using the same standards as conventional clinical electroencephalography (Cert. Number:302 AFBZX00079000, Class II, EEG)^1^ (available on 11th October 2023) The electrode sheet of this device can be easily attached to the patients’ forehead skin, eliminating the discomfort typically associated with headgear devices. It weighs only 27 g and has a curved shape that fits the user’s forehead. A wireless communication interface based on the Bluetooth Low-Energy protocol was used to control the device. This device has a high voltage resolution of up to 24 bits (22 nV/LSB) and low input reference noise of 1μVpp. Multichannel EEG signals can be recorded at a sampling rate of 250 Hz in three channels (center, left, and right). The thickness of the electrode sheet is less than 50 μm, the elasticity is less than 200%, and the moisture permeability is 2,700 g/m^2^/day. The sheets were manufactured using a cost-effective screen-printing process using a biocompatible gel and an Ag-based material on an elastic base. The biocompatibility of the conductive and non-conductive gels was evaluated using the ISO 10993 standard for skin sensitization, irritation, and *in vitro* cytotoxicity (17).

### 2.4. EEG measurements

EEG measurements were conducted on any one day from patients’ admission to the day before surgery. During the EEG measurement, we affixed the aforementioned electroencephalography sheet to the patient’s forehead while they were seated in a chair. We then asked the patient to close their eyes and relax, after which the EEG recording was initiated. When the EEG signals were properly measured and the hum noise was less than 5 μV, the actual measurement was started. If the hum noise was higher, the measuring site were adjusted until the noise was below the baseline, then the measurement was started. EEG data of the patients were recorded preoperatively upon confirming that their mental status has not changed. The recorded EEG data during a 2-min-eyes-closed resting state, supervised by the examinators were analyzed in this study.

### 2.5. EEG signal processing and analysis

We analyzed 2-min-resting-eyes-closed EEG data on three channels, applied a 4–75 Hz band pass filter (18) and a 60 Hz notch filter to remove the powerline interference. The power spectrum density (PSD) (19) was calculated for each channel, and the mean PSD was calculated for each frequency band (theta 4–8 Hz, alpha 8–13 Hz, beta 13–30 Hz, gamma1 30–59 Hz, gamma2 61–75 Hz). The above 5 × 3 = 15 values and their respective ratios were used as features for machine-learning analysis. All patients’ 2-min-data were divided into 2-s-segments for a total of 60 samples for each patient. In this study, for the purpose of application to a simple screening test, the entire 2-min resting eye-closure recordings of all subjects were used for analysis, without arbitrary artifact removal that would require specialized techniques, yielding no missing data. To avoid data leakage from the same individual, we partitioned the entire dataset at a 4:1 ratio into training and testing data, ensuring no overlap of same individual EEG data between the two groups. The training data was then further divided into four groups to perform group *k*-fold cross-validation (*k* = 4) on the training data to train the prediction model. Lastly, we evaluated the trained model by using the testing data. The standard machine-learning models, PyCaret frameworks^2^ (available on 31th Aug 2023) were applied by training dataset, and “Extra Trees Classifier” model was adopted because of the highest performance among those trainings. “Extra Trees classifier” model was recently reported promising results in the fields of EEG and neurophysiological studies (20, 21). Based on the developed prediction model, feature importance was investigated to examine the contribution of the parameters to the prediction. Python (version 3.8.13) was used for these analyses, and PyCaret (version 2.3.10) was used for the cross-validation and machine-learning analysis. Technical details were shown in the Supplementary material.

### 2.6. Statistical analyses

Regarding demographic data, age (continuous, parametric) comparisons between patients with and without postoperative delirium (POD) were conducted using Student’s *t*-test. The chi-square test was employed for analyzing sex differences (categorical), ratios of benzodiazepines or anticholinergics users (categorical) and surgical procedures (categorical), while the Mann–Whitney *U* test was used to compare Mini-Cog scores (nonparametric) and treatment days (nonparametric) in the ICU. In the EEG analysis, PSD (continuous, parametric) was compared between patients with and without POD using Student’s *t*-test, and Bonferroni corrections were applied based on the number of electrodes (three channels) and frequency bands (five frequencies). All thresholds for significance were set at *p* = 0.05.

## 3. Results

### 3.1. Demographic data analysis

**Figure 1:**
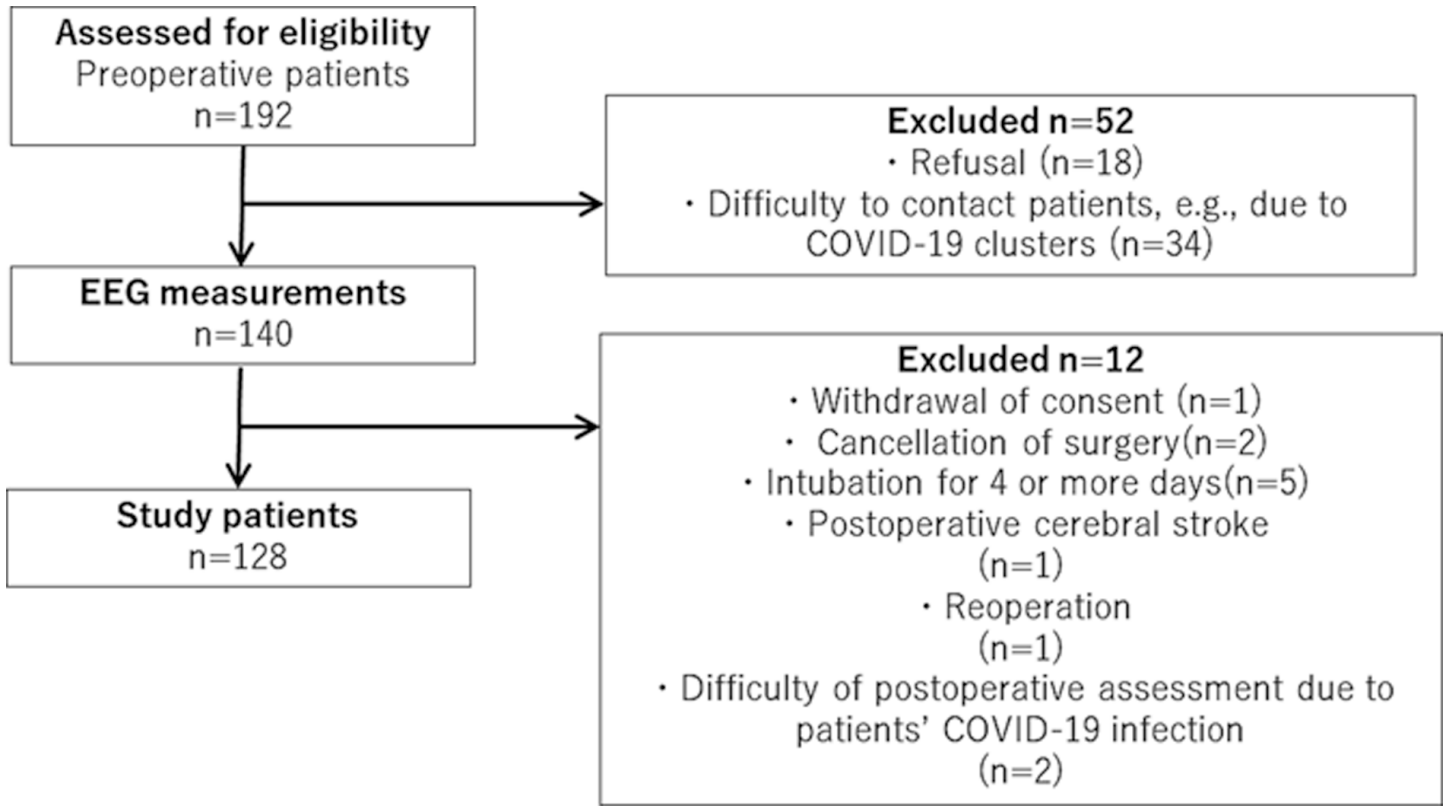
Flowchart of patient recruitment. During the study period, there were 192 eligible patients, of whom 128 were finally included in this study.

During the study period, there were 192 eligible patients, of whom 128 were finally included in this study, as shown in Figure 1. The demographic and clinical information of all the study participants is summarized in Table 1. EEG measurements and Mini-cog tests were conducted on any one day from patients’ admission to the day before surgery, with the average being 3.1 ± 2.8 (mean ± SD) days prior to surgery. A total of 128 patients were included in the study, of which 47 had POD, which is an incidence of 36.7%. The incidence of delirium was 37.5% in male and 35% in female patients. The onset of postoperative delirium was identified on 2.2 ± 1.4 (mean ± SD) days postoperatively. No significant differences were found between the study patients with and without POD in terms of age, sex, Mini-Cog scores, duration of ICU stay, ratios of benzodiazepines or anticholinergics users, and surgical procedures. The total number of surgical procedures exceeded 100% because some cases had comorbidities and were approached with more than one surgical procedure.

**Table 1:** Demographic and clinical data of all study patients after surgery.

	Delirium positive	Delirium negative	Value of *p*
Number of subjects	47	81	
gender(male/female)	33/14	55/26	0.394
Age (years ± SD)	70.9 ± 9.6	67.7 ± 10.9	0.096
Mini-cog (scores ± SD)	4.0 ± 1.1	4.2 ± 0.9	0.184
ICU stay (days ± SD)	3.1 ± 1.6	2.9 ± 1.7	0.48
Benzodiazepines users	6(12.8%)	7(8.6%)	0.547
Anticholinergics users	10(21.3%)	22(27.2%)	0.521
surgical procedures	CABG:17(36.2%)	CABG:32(39.5%)	0.853
	GR:7(14.9%)	GR:13(16.0%)	0.996
	Valve:33(70.2%)	Valve:47(58.0%)	0.237

ICU, Intensive care unit; CABG, coronary artery bypass graft; GR, graft replacement; Valve, Valve surgery.

### 3.2. Power spectrum density estimated from EEG signals between study patients with and without POD

**Figure 2:**
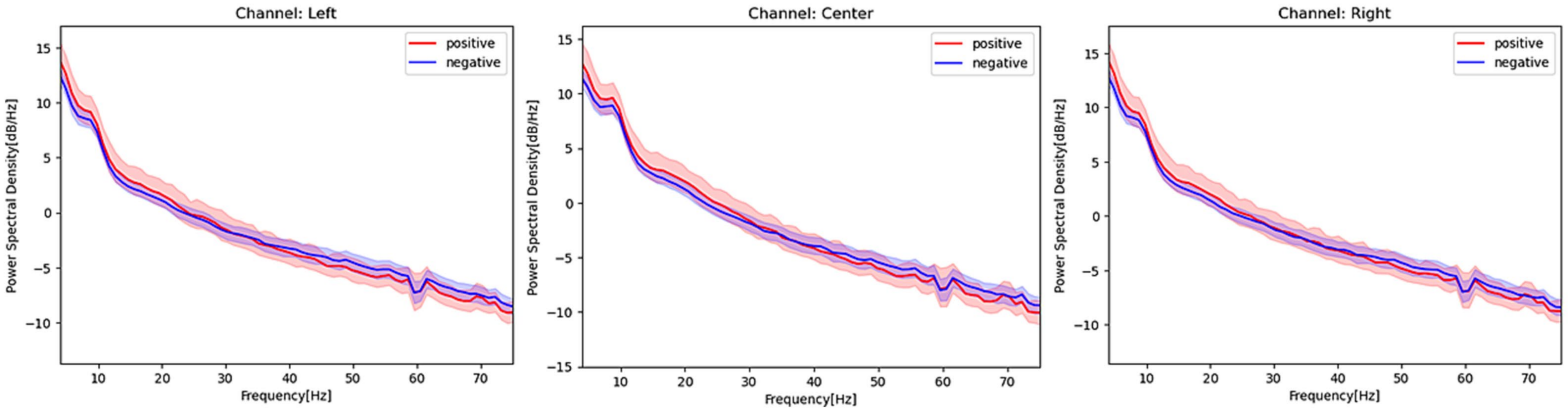
Power spectrum density estimated from EEG signals in patients with and without postoperative delirium. Red activities in the figure represent the power spectrum density, with a 95% confidence interval, of patients with postoperative delirium. Blue activities represent the power spectrum density of patients without postoperative delirium at each EEG channel. The frequency range covered in this figure is 4–75 Hz, and a notch filter at 60 Hz has been applied.

Figure 2 shows the PSD of patients with and without POD in each channel at 4–75 Hz, notch filtered at 60 Hz. Patients with POD exhibited slightly higher PSD values at relatively slow frequencies (i.e., less than 30 Hz) but were slightly lower in the gamma band in opposite directions at each channel. A comparison of the PSDs between patients with and without POD in each frequency band is presented in Table 2. Patients with POD were characterized by significantly lower activity in the PSD gamma band.

**Table 2:** Comparison of power spectrum density in patients with and without postoperative delirium for each channel and frequency.

Channel-frequency	*t*-value	Value of *p*
Center-theta	3.883	0.0001*
Center-alpha	3.294	0.00099*
Center-beta	4.391	0.00001*
Center-gamma1	−9.368	<0.00001*
Center-gamma2	−8.746	<0.00001*
Right-theta	3.974	0.000071*
Right-alpha	1.909	0.0563
Right-beta	0.135	0.8923
Right-gamma1	−9.762	<0.000001*
Right-gamma2	−7.381	<0.000001*
Left-theta	3.215	0.0013*
Left-alpha	1.512	0.1305
Left-beta	−0.08	0.9364
Left-gamma1	−13.03	<0.000001*
Left-gamma2	−10.29	<0.000001*

^*^Significant after Bonferroni correction. A *t*-value greater than zero indicates higher power spectrum density in patients with postoperative delirium compared to patients without delirium. Asterisks indicate significant differences after Bonferroni correction.

### 3.3. Prediction of patients with POD based on machine-learning analysis and feature importance assessment

**Figure 3:**
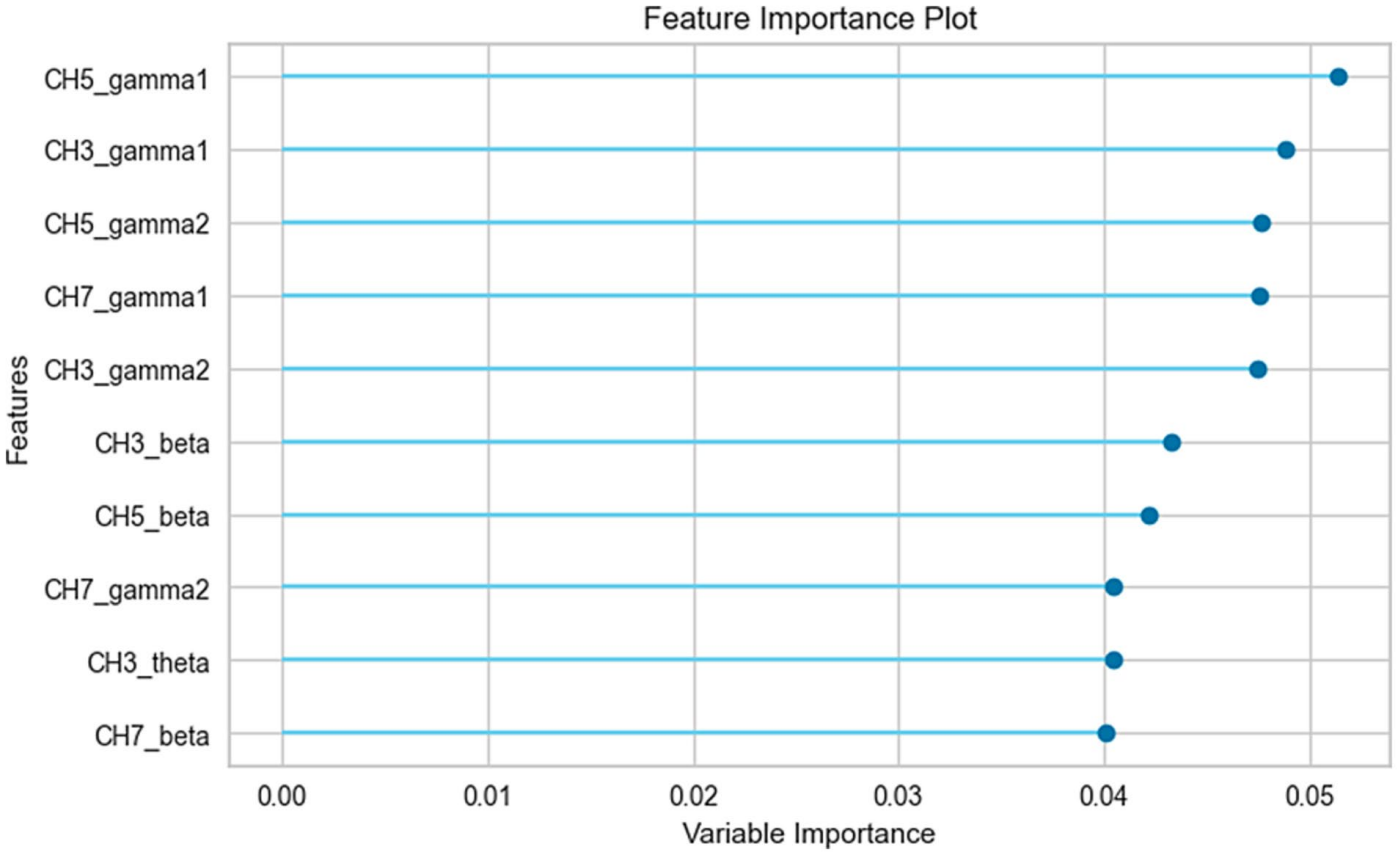
Feature importance analysis of the prediction model. The top 10 parameters, ranked by their contribution to the prediction model, are shown in the figure. CH3, center channel; CH5, right channel; CH7, left channel.

Patients with POD were predicted with an accuracy of 86% and an area under the receiver operating characteristic curve (AUROC) of 0.93, utilizing preoperative EEG oscillations. Figure 3 showcases the top ten parameters contributing the most to this prediction. Notably, the five most influential parameters contributing to the prediction were associated with gamma activity.

## 4. Discussion

This study provides a precise machine-learning algorithm for predicting the onset of POD in patients undergoing cardiovascular surgery, utilizing preoperative EEG signals obtained through a patch-type portable device. Moreover, this study provides new insights into the objective and biological characteristics associated with vulnerability to delirium.

Several prospective studies (22–24) have investigated the prediction of PODs in patients undergoing cardiovascular surgeries using a substantial array of preoperative clinical parameters. These studies reported an area under the curve (AUC) values ranging from 0.74 to 0.83, which were marginally lower than the findings in this study. In this study, a notable advantage was observed in achieving precise predictions solely through the use of a single modality, EEG, without the need for incorporating additional clinical parameters. In the future, even greater accuracy in predicting postoperative delirium could potentially be attained by integrating the clinical parameters and EEG features investigated in this study. A few studies that analyzed preoperative EEG measurements in relation to postoperative delirium reported no significant differences in preoperative relative delta power between patients with and without POD (23). These results could be attributed to the fact that a previous study (25) focused on slow activity in the delta band. However, the EEG device used in this study has the functional property of precisely capturing high-frequency activities (18), enabling precise analysis in the high-frequency range, distinctive gamma activities shown in the PSD, and feature importance analyses. Increased EEG gamma activity was associated with conscious awareness (26), then, relatively lower gamma activities in the preoperative patients with POD could be related to lower conscious awareness which potentially reflected vulnerability to delirium. Low gamma activity of the perioperative frontal EEG was reported to be associated with the onset of POD (27), which is consistent with this study, suggesting that gamma EEG activity could be a promising neurophysiological feature that predicts the onset of POD.

The proposed approach has several advantages. The predictive EEG signals were obtained in only 2 min and could be automatically analyzed using a machine-learning algorithm, allowing rapid screening. When this approach becomes available in usual clinical settings, it will help predict and screen a large number of patients in several settings, including inpatient, outpatient, emergency room, nursing home, and potentially the patient’s house, which is never possible with conventional EEG (12). This simple EEG measurement and automated analysis could support the identification of high-risk groups for POD and reduce the risk of developing delirium through intensive preoperative intervention (4), yielding a profound impact on clinical practice.

One of the limitations of this study is that it was conducted at a single Japanese university hospital. Thus, a validation study with multicenter institutions is needed to make our results more generalizable. The incidence of postoperative delirium after cardiac surgery ranges from 4.1 to 54.9% (28, 29), and the incidence of POD in this study was 36.7%, which was within the reported range. Furthermore, this study only focused on postoperative deliriums after cardiovascular surgeries, thus our results hold limitations in extending speculation to delirium based on other factors. Next, delirium status was not scored; thus, EEG features related to delirium severity were not examined in this study. In addition, no significant differences were found between the patients with and without POD on a brief cognitive test; however, the possibility of minor differences in more precise cognitive measurements could not be excluded.

## 5. Conclusion

This study revealed that postoperative delirium after cardiovascular surgery can be precisely predicted from preoperative EEG oscillations measured using a patch-type portable device with machine-learning analysis. The proposed algorithm can be applied to general hospitals without advanced equipment and expert knowledge, and therefore could be implemented in clinical practice to identify patients at high risk of postoperative delirium. By enabling the targeted application of intensive interventions, this approach has the potential to effectively reduce the incidence of postoperative delirium.

## Data availability statement

The original contributions presented in the study are included in the article/Supplementary material, further inquiries can be directed to the corresponding author.

## Ethics statement

The studies involving humans were approved by the ethics committee of Osaka University Hospital. The studies were conducted in accordance with the local legislation and institutional requirements. The participants provided their written informed consent to participate in this study.

## Author contributions

MH: Conceptualization, Writing – original draft, Writing – review & editing, Data curation, Formal analysis, Funding acquisition, Investigation, Methodology, Project administration, Resources, Software. YM: Conceptualization, Formal analysis, Writing – review & editing. CN: Data curation, Writing – review & editing. HM: Supervision, Writing – review & editing. TW: Supervision, Writing – review & editing. ST: Supervision, Writing – review & editing. RI: Supervision, Writing – review & editing. SM: Supervision, Writing – review & editing. MI: Supervision, Writing – review & editing. TU: Supervision, Writing – review & editing.

## Footnotes

^1^ https://www.info.pmda.go.jp/ygo/pack/651319/302AFBZX00079000_1_01_02/302AFBZX00079000_1_01_02?view=body

^2^ https://www.pycaret.org
