# Supplementary material for: Predicting postoperative delirium after cardiovascular surgeries from preoperative portable electroencephalography oscillations
Source: Front Psychiatry. 2023 Nov 14;14:1287607. doi: 10.3389/fpsyt.2023.1287607 (PMC10682064; doi:10.3389/fpsyt.2023.1287607)
Supplement: Supplementary file 1 [file Data_Sheet_1.docx]

**Supplementary Figure. Image of Patch-type portable EEG device**

The elastic electrode sheet was shown in the left figure. The sheet was connected to the device with a plug connector. The EEG device sensor can be easily attached on the patient’s forehead without any binding method in the right figure. The reference electrode was attached to the patients’ mastoid process.


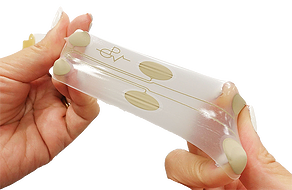

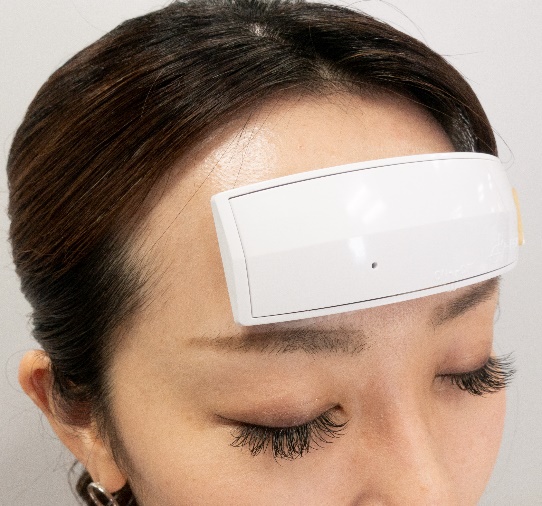


**Technical details**

PyCaret is a low-code machine learning library that simplifies the entire process from data preprocessing to model training and validation. Users can easily set parameters to efficiently perform tasks like dataset splitting, model training, and validation.

The Extra Trees Classifier is one of the ensemble learning methods available in the PyCaret library. This model fits randomized decision trees to various sub-samples of the dataset and averages their predictions to improve predictive accuracy and control overfitting. PyCaret utilizes the Extra Trees Classifier from scikit-learn. More information was available at the following website (available on 11^th^ Oct, 2023). (https://scikit-learn.org/stable/modules/generated/sklearn.ensemble.ExtraTreesClassifier.html)

The importance of a feature is computed as the (normalized) total reduction of the criterion brought by that feature. It is also known as the Gini importance. More information was available at the following website (available on 11th Oct, 2023).

(https://scikit-learn.org/stable/modules/generated/sklearn.tree.ExtraTreeClassifier.html)

We employed power across general frequency bands as features, and given two features, a and b, the ratio a/b was computed. Interaction threshold was set at 0.01 as default value in PyCarat. More information was available at the following website (available on 11th Oct, 2023).

(https://github.com/pycaret/pycaret-docs/blob/main/get-started/preprocessing/feature-engineering.md)”
